# Fructose: A Key Factor in the Development of Metabolic Syndrome and Hypertension

**DOI:** 10.1155/2013/682673

**Published:** 2013-05-25

**Authors:** Zeid Khitan, Dong Hyun Kim

**Affiliations:** ^1^Marshall University's Joan C. Edwards School of Medicine, 1600 Medical Center Drive, Huntington, WV 25701-3655, USA; ^2^Department of Medicine, Marshall University Joan Edwards School of Medicine, 1600 Medical Center Drive, Huntington, WV 25701-3655, USA

## Abstract

Diabetes mellitus and the metabolic syndrome are becoming leading causes of death in the world. Identifying the etiology of diabetes is key to prevention. Despite the similarity in their structures, fructose and glucose are metabolized in different ways. Uric acid, a byproduct of uncontrolled fructose metabolism is known risk factor for hypertension. In the liver, fructose bypasses the two highly regulated steps in glycolysis, glucokinase and phosphofructokinase, both of which are inhibited by increasing concentrations of their byproducts. Fructose is metabolized by fructokinase (KHK). KHK has no negative feedback system, and ATP is used for phosphorylation. This results in intracellular phosphate depletion and the rapid generation of uric acid due to activation of AMP deaminase. Uric acid, a byproduct of this reaction, has been linked to endothelial dysfunction, insulin resistance, and hypertension. We present possible mechanisms by which fructose causes insulin resistance and suggest actions based on this association that have therapeutic implications.

## 1. Background

Type 2 diabetes mellitus is characterized by hyperglycemia, insulin resistance, and an impairment in insulin secretion. In the late nineteenth century, William Osler described diabetes as a rare disorder more likely to develop in obese people and patients with gout. He estimated its prevalence as approximately two to nine cases per 100,000 population in the USA and Europe being more common in the latter [1]. Diabetes, one of the leading causes of death in the United States, affects over 200 million people worldwide. The estimated prevalence of diabetes among adults in the United States ranges from 4.4 to 17.9 percent [2]. The community-based Framingham Heart Study, in a predominantly non-Hispanic white population, found a doubling in the incidence of type 2 diabetes over the last 30 years [3]. Identifying the etiology of type 2 diabetes is a key to its prevention. Obesity and intra-abdominal fat accumulation induce insulin resistance [4]. Studies have documented high rates of type 2 diabetes in the absence of classic obesity [5]. This suggests that other risk factors besides obesity might play a role in the epidemic of type 2 diabetes.

## 2. Fructose: Sources and Metabolism

**Figure 1:**
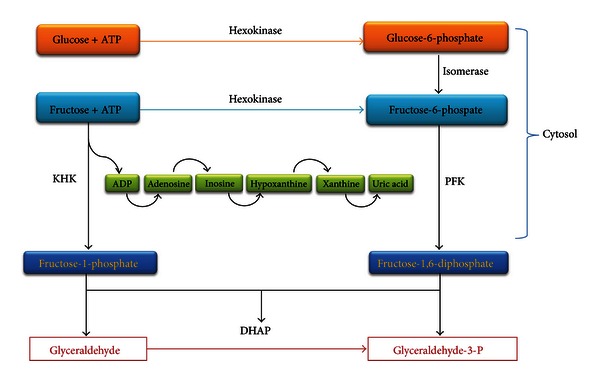
Fructose metabolism. Fructose is primarily metabolized to fructose-1-phosphate by KHK due to its lower Km for fructose compared with hexokinase. Uncontrolled consumption of ATP leads to intracellular phosphate depletion and activation of AMP deaminase leading to the increased production of uric acid. Fructose-1-phosphate is further metabolized by aldolase B and triokinase to glyceraldehyde-3-phosphate.

**Figure 2:**
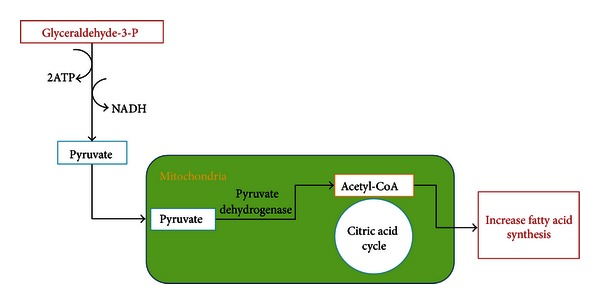
Role of fructose in lipogenesis. Glyceraldehyde-3-P continues downstream in the glycolysis pathway forming pyruvate which enters the mitochondria and is further metabolized to acetyl-CoA by pyruvate dehydrogenase. Acetyl-CoA enters the citric acid cycle by combining with oxaloacetate to form citrate. In the well fed state, citrate can be transported to the cytosol, providing CoA necessary for lipogenesis.

Fructose is a simple sugar present in fruits and honey and is responsible for their sweet taste. However, the major source of fructose worldwide is sucrose or table sugar, which is derived from sugar cane and sugar beets. It is man-made, first developed in New Guinea and in the Indian subcontinent and was a rare and expensive commodity that was introduced into Europe via Venice, Italy, and other trading ports during the Middle Ages. Sucrose is a disaccharide that is comprised of fructose and glucose. After ingestion, sucrose is degraded in the gut by sucrase, releasing fructose and glucose that are then absorbed. In addition to sucrose, the other major source of fructose is high fructose corn syrup (HFCS), which was introduced in the early 1970s as an additional sweetener. HFCS consists of fructose and glucose mixed in a variety of concentrations, but most commonly as 55% fructose and 45% glucose. In the United States, HFCS and sucrose are the major sources of fructose in the diet, and HFCS is a major ingredient in soft drinks, pastries, desserts, and various processed foods [6, 7]. Despite the similarity in their chemical structures, fructose and glucose are metabolized in completely different ways and utilize different GLUT transporters [8]. In the liver, fructose bypasses the two highly regulated steps of glycolysis, catalyzed by glucokinase/hexokinase and phosphofructokinase both of which are inhibited by increasing concentrations of their byproducts. Instead, fructose enters the pathway at a level that is not regulated and is metabolized to fructose-1-phosphate primarily by fructokinase or ketohexokinase (KHK) (Figures 1 and 2). Fructose may also be metabolized by hexokinase; however, the Km for fructose is much higher than glucose, and hence minimal amounts of fructose are metabolized via this pathway [6]. Fructokinase has no negative feedback system, and ATP is used for the phosphorylation process. As a result, continued fructose metabolism results in intracellular phosphate depletion, activation of AMP deaminase, and uric acid generation which is harmful at the cellular level [6, 9, 10].

Fructose-1-phosphate is subsequently converted to dihydroxyacetone-phosphate and D-glyceraldehyde by the action of the aldolase B. D-glyceraldehyde is phosphorylated and continued downstream in the glycolysis pathway to form pyruvate. Two of the most energetic reactions of all organophosphates are in the pathway of fructose metabolism, catalyzed by phosphoglycerate and pyruvate kinases. Two ATP molecules as well as free energy, approximately 12 kcal/mole, are released [12]. Fructose controls the activity of glucokinase, the principle enzyme of glucose metabolism in the liver. Fructose is a potent and acute regulator of liver glucose uptake and glycogen synthesis. Inclusion of catalytic quantities of fructose in a carbohydrate meal improves glucose tolerance. This improvement is primarily mediated by the activation of hepatic glucokinase resulting in improved liver glucose uptake [13]. 

Uric acid, a byproduct of fructose degradation, stimulates KHK expression through the activation of the transcription factor ChREBP, which in turn results in the transcriptional activation of KHK by the binding to a specific sequence within its promoter [14]. Uric acid inhibits endothelial NO both *in vivo* and *in vitro*, [15] and directly induces adipocyte dysfunction [16]. Serum uric acid increases rapidly after ingestion of fructose, resulting in increases as high as 2 mg/dL within 1 hour [17–19]. Uncontrolled fructose metabolism leads to postprandial hypertriglyceridemia, which increases visceral adipose deposition. Visceral adiposity contributes to hepatic triglyceride accumulation, protein kinase C activation, and hepatic insulin resistance by increasing the portal delivery of free fatty acids to the liver [20]. 

A number of other furanose sugars can also act as KHK substrates [21]. KHK is expressed as two isoforms, KHK-C and KHK-A. KHK-C is primarily expressed in the liver, kidney, pancreas, and duodenum, while KHK-A is expressed more widely including adipose tissue, heart, and the adrenal gland [22]. The exact biologic function of KHK-A is unknown. KHK-A has a higher Km for fructose (7 mmol/L) than does KHK-C (0.8 mmol/L) suggesting that it phosphorylates fructose poorly at physiological concentrations [23]. Recently, it was found that adiposity and metabolic syndrome were prevented in mice lacking both KHK isoforms but exacerbated in mice lacking KHK-A [24]. It was also demonstrated that neither KHK isoform is required for normal growth and development in rats [25]. Serum leptin, triglycerides, and fasting blood glucose levels are higher in humans placed on a high fructose diet for four weeks compared with those on a starch-based diet [26].

Deficiency of KHK-C, an autosomal recessive inborn error of metabolism, results in essential fructosuria with an estimated incidence of 1 : 130,000 [27]. This condition was first recognized in 1876 [23]. It is an anomaly rather than a disease, since it does not lead to any outward signs or symptoms. Most cases of fructosuria have been described in Jewish families [28]. It has no metabolic or morbid manifestations other than having transient fructosuria after meals containing either sucrose or fructose. This condition used to be detected during routine medical examination when tests based on reducing properties of glucose like Benedict's solution and Clini test were used to diagnose diabetes. These tests have since been replaced by the more specific glucose oxidase method which does not react with fructose; therefore, patients with essential fructosuria are no longer being identified [28]. Moreover, the lack of treatment consideration and counseling for affected individuals and their family members, the absence of screening recommendations, and the lack of serum KHK assay makes it difficult to identify subjects with this anomaly. This is in contrast to hereditary fructose intolerance, a disease characterized by the deficiency of fructose-1-phosphate aldolase which has significant metabolic and developmental complications that manifest themselves as early as in the neonatal period. In a well-characterized family, in which three of eight siblings have fructosuria, all affected individuals are compound heterozygotes for the mutations Gly40Arg and Ala43Thr [29].

## 3. Epidemiological Evidence

Since 1970, the total availability of sugars has dramatically increased. Comparison of the 1977–1978 NFCS analysis with the analyses of NHANES for the period 1999–2004 indicated that, over the intervening period, mean individual intake of total fructose increased by *∼*32% [30]. HFCS now represents nearly 50% of caloric sweeteners use in the United States [30, 31]. Increased total fructose consumption has been implicated in the development of the obesity epidemic in the United States with the consumption of HFCS increased >1000% between 1970 and 1990, far exceeding the changes in intake of any other food or food group [32]. 

Several reviews have concluded that intake of both fructose and HFCS by children and adults was associated with an increased risk of obesity and metabolic syndrome [33–37]. However, not all published meta-analyses have reported a statistically significant link [38–41]. Recently, Sievenpiper and colleagues concluded in a meta-analysis of controlled feeding trials that fructose does not cause weight gain when substituted for other carbohydrates in isocaloric trials [42]. This was criticized on the basis of fructose causing weight gain by altering appetite resulting in increased food intake, by inducing leptin resistance and by a direct effect on the brain indicating that isocaloric trials do not show a difference in weight gain between groups. Moreover, the use of weight gain as a marker is subject to debate, since other fructose effects, beyond body weight as central fat accumulation and insulin resistance, can be more important [43].

In children, intake of artificially sweetened beverages was found to be positively associated with adiposity [44]. A prospective cohort analyses conducted from 1991 to 1999 among nondiabetic women in the Nurses' Health Study II concluded that higher consumption of sugar-sweetened beverages was associated with greater magnitude of weight gain and an increased risk for the development of type 2 diabetes [45]. On the other hand, a recent analysis of NHANES 1999–2006 databases comparing data from >25,000 subjects showed no relation between daily fructose intake and the indicators of metabolic syndrome, uric acid, and BMI [46]. This study was based on a snapshot of one- or two-days recalls. Thus, the intake data may not truly represent the long term, consistent consumption of food. Moreover, due to no fructose intake data being available in NHANES databases and a lack of fructose content data for many food items in the USDA National Nutrient Database for Standard Reference, the fructose intake of individuals was indirectly estimated using several databases.

Interpretation of cross-sectional studies examining the relationship of sugar intake to obesity can be misleading due to the fact that subjects who become obese may well reduce their sugar intake, since sugar is widely recognized to cause weight gain. Therefore, examining the relationship of sugar to obesity is best performed with well controlled, long-term longitudinal studies. It is also important to recall that leptin resistance leading to liporegulatory failure, and, subsequently, insulin resistance can be perpetuated once obesity and intracellular lipid accumulation are manifest, especially in sites other than adipose tissue including pancreatic B cells and cardiomyocytes. Thus, reducing fructose intake may not fully reverse insulin resistance and diabetes. This further adds to the complexity and clouds the interpretation of these epidemiological data [6, 47, 48].

The relationship between fructose intake and hypertension was also examined in several clinical studies. In a randomized controlled trial, high dose fructose (200 gm/24 h) increased ambulatory blood pressure and elevated fasting insulin levels. In this study, allopurinol prevented the increase in mean arterial blood pressure [49]. In an analysis of the NHANES 2003–2006 data, fructose intake, in the form of added sugar, was independently associated with higher blood pressure levels [50]. Fructose consumption in the form of sugar-sweetened beverages was associated with hypertension and elevated uric acid level in USA adolescents [51] but not in adults [52]. Uric acid may raise systemic blood pressure by increasing inflammation, activating the renin-angiotensin system, and decreasing nitric oxide production contributing to renal vasoconstriction that results in salt insensitive hypertension [53]. Persistent vasoconstriction may contribute to arteriosclerosis and the subsequent development of salt-sensitive hypertension, even if the hyperuricemia is corrected [54]. This may explain the different results in the preceding two studies that looked at two different age groups. A recent meta-analysis of controlled feeding trials found that isocaloric substitution of fructose with other carbohydrates did not adversely affect blood pressure in humans suggesting that there is a need for long-term and large trials to clarify these findings [40].

Few studies have examined the role of naturally occurring sugars, for example, 100% fruit juice, in the origin of obesity and related end points. It is believed that fructose from natural sources can be less harmful because the presence of additional nutrients and antioxidants. On the other hand, crystalline fructose, as in table sugar, and HFCS are regarded as less safe, since glucose present in these sugars can accelerate fructose absorption. 100% fruit juice consumption among USA adults is associated with lower insulin resistance [55] and lower odds of obesity and metabolic syndrome. Obesity remained an independent factor after adjusting to other lifestyle factors. What is interesting in this study is that 100% juice consumers had significantly higher white milk intake which, on its own and among other dairy products, has been shown to enhance weight loss [43, 56, 57].

There is mounting evidence from studies looking at the association between fructose and obesity and metabolic syndrome that primary fructose malabsorption in children was negatively associated with obesity [58]. In obese African-Americans, high rates of fructose malabsorption were associated with reduced liver fat thought to be protective against fatty liver disease [59].

In conclusion, it is evident that there is a need for clinical trials with variable amounts of fructose intake to determine effects on metabolic outcomes rather than depending on meta-analyses of existing studies of mixed design and duration.

## 4. Effect of Fructose on Adipocyte Differentiation

Adipocyte development in mice and humans follows a well-defined pathway that begins with a common stem cell mediated adipocyte regeneration and is referred to as adipogenesis [60]. The first step of adipogenesis is the generation and commitment of mesenchymal stem cells (MSCs) to adipocyte lineage. The effect of fructose-mediated renin-Ang II activation is on the stages of cell differentiation (“commitment”) and involves local and systemic effects. 

**Figure 3:**
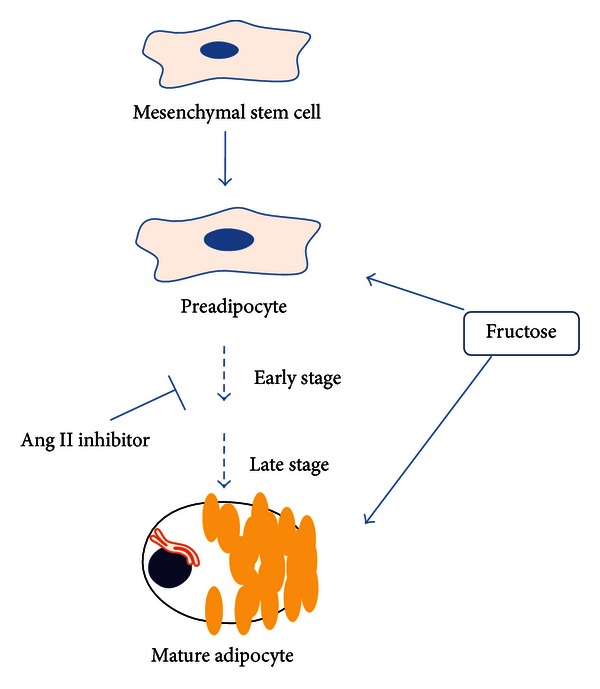
Overview of stages of adipocyte differentiation and the impact of angiotensin II inhibitors and elevated fructose levels.

Figure 3 shows the mechanism of Ang II inhibition during adipogenesis. MSCs or preadipocytes differentiate into lipid-laden and insulin-sensitive adipocytes [61]. 

Briefly, the stages of adipocyte differentiation are affected by increases in the levels of fructose and Ang II which cause MSC-derived adipocyte growth arrest, clonal expansion, and early differentiation. Ang II blockade can prevent terminal differentiation leading to the development of the mature adipocyte phenotype [62, 63]. 

Normal diets result in the activation of PPAR*γ* followed by adipose expansion through adipocyte hyperplasia, resulting in an increased number of new preadipocytes. The resulting adipocytes are small in size and effectively store lipids, thereby reducing lipotoxicity in the liver and adipose tissue and release adiponectin [64, 65]. Activation of these genes leads to repartitioning of lipids resulting in an increased triglyceride content of adipose tissue, a lowered free fatty acid content in circulation and availability for liver and muscle use, thereby improving insulin sensitivity. Methylisobutylxanthine (MIX), an phosphodiesterases inhibitor, increases intracellular cAMP, activating adipocyte differentiation in a PKA-independent manner [66]. MIX also increased the expression of C/EBP-*β*, required for the subsequent expression of PPAR-*γ* [62].

Although, the mechanisms by which fructose controls adipogenesis *in vivo* are largely unknown, there are a number of candidates that mediate adipocyte differentiation in culture and are thought to control adipocyte accumulation and function *in vivo*. Two main factors fit this criterion: (1) high fructose diets, (2) increases in ROS. They have been implicated as the link between adipogenesis and metabolic diseases including T2DM.

Recent studies demonstrated that the induction of oxidative stress by high fructose or glucose increased NAD(P)H oxidase and the mitochondrial respiratory chain which is associated with diabetic complications [67]. Therefore, fructose diets may lead to adipocyte differentiation associated with adipocyte dysfunction and formation of adipocytes external to normal adipocyte depots, that is, muscle, liver, and pancreas leading to advanced diabetic complications.

**Figure 4:**
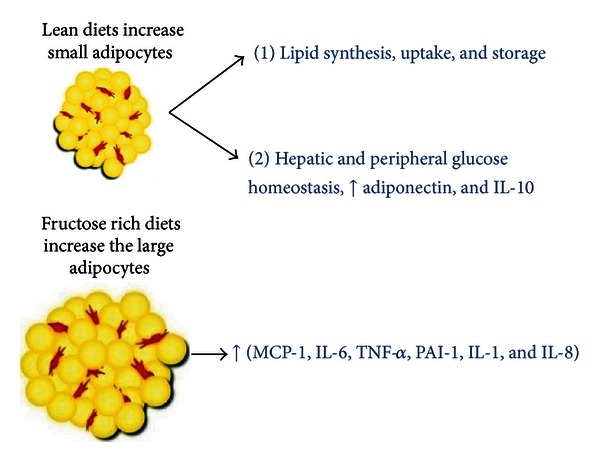
Enlargement of adipocytes causes alterations in secretion of adipokines. Under normal conditions, adipocyte is a site of lipid synthesis, uptake, and storage. Secreted adipokines function as endocrine, paracrine, or autocrine mediators. Increased adipocyte size can lead to deleterious alterations in insulin sensitivity caused by a decrease in adiponectin secretion and the induction of inflammatory mediators. Modified from [11].

The fructose-mediated increase in ROS via activation of the adipocyte renin-Ang II system may lead to adipocyte dysfunction and insulin resistance. Adipose tissue is a key endocrine organ, the function of which, via interaction with the vascular endothelium system, regulates lipid uptake, storage, synthesis, and secretion of paracrine and autocrine factors that regulate insulin sensitivity. However, fructose-mediated vascular dysfunction may have a negative effect on adipocyte function and the secretion of anti-inflammatory molecules such as adiponectin, IL-1, and IL-10. The glucose or fructose-mediated decrease in vascular function increases adipocyte size resulting in decreased levels of adiponectin, but increased levels of MCP-1, IL-6, and TNF-*α* that have systemic effects on *β* cells (Figure 4).

**Figure 5:**
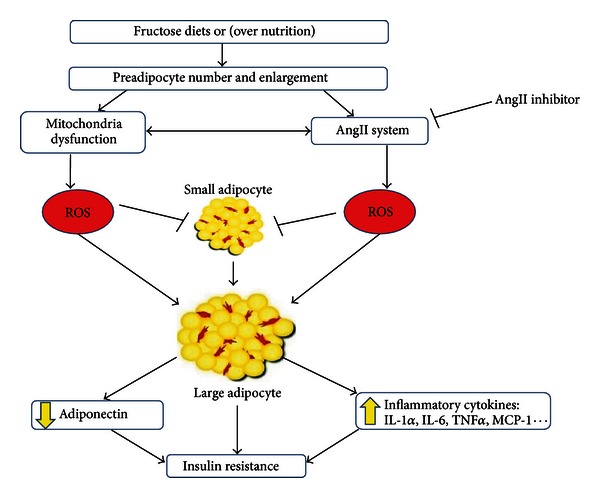
Molecular mechanisms by which fructose diets, inactivity, and gluttony increase preadipocyte number and enlargement via increases in ROS generated by the renin-AngII system and mitochondrial dysfunction leading to obesity, insulin resistance, and diabetes. Hyperglycemia results in increased ROS production within the mitochondria via a number of mechanisms including a reduction in the glutathione/glutathione disulfide ratio. ROS generation mediates a proinflammatory cascade resulting in increase of adipogenesis, release of inflammatory cytokines, and decrease in adiponectin leading to insulin resistance.

The adipocyte-mediated increase in adipokine release plays a critical role in the regulation of blood pressure (angiotensinogen), vascular haemostasis, and angiogenesis. The release of these cytokines by adipocytes suggest that fructose-mediated diabetes may be related to systemic effects which include altered adiposity and insulin resistance. Adipocyte dysfunction occurs as a consequence of chronic overfeeding of fructose leading to adipocyte enlargement and inflammation and mitochondrial dysfunction (Figure 5).

Mitochondria play an important role in adipocyte differentiation and function [68]. During the early stages of preadipocyte development, an increased number of mitochondria are required, resulting in small mature adipocytes, highly sensitive to insulin, and secreting high levels of adiponectin [69]. By contrast, mitochondrial dysfunction has also been linked to T2DM complications in fructose diets. The results of impaired mitochondrial function include increased FFA levels resulting in the accumulation of mitochondrial products including fatty acyl coenzyme A (CoA) and reduced insulin sensitivity [69].

In summary, fructose diets, inactivity, and gluttony results in adipocyte expansion with the resultant detrimental perturbations in the renin-Ang II system and in mitochondria, both of which undergo cellular changes that result in the increased generation of ROS and TNF-*α*, IL-1, and IL-6 and a decrease in adiponectin levels. Adiponectin is synthesized and released only by the adipocyte and has an essential role in vascular and renal function.

Increases in adipocyte release of adiponectin inhibits both the expression of hepatic gluconeogenic enzymes and the rate of endogenous glucose production in diabetic mice [70]. In adiponectin transgenic mice, adiponectin reduced the expression of phosphoenolpyruvate carboxylase and glucose-6-phosphatase, which are associated with elevated phosphorylation of hepatic AMPK and decreased glucose production [70, 71].

## 5. Prohypertensive Effects of Fructose and Putative Mechanisms

**Figure 6:**
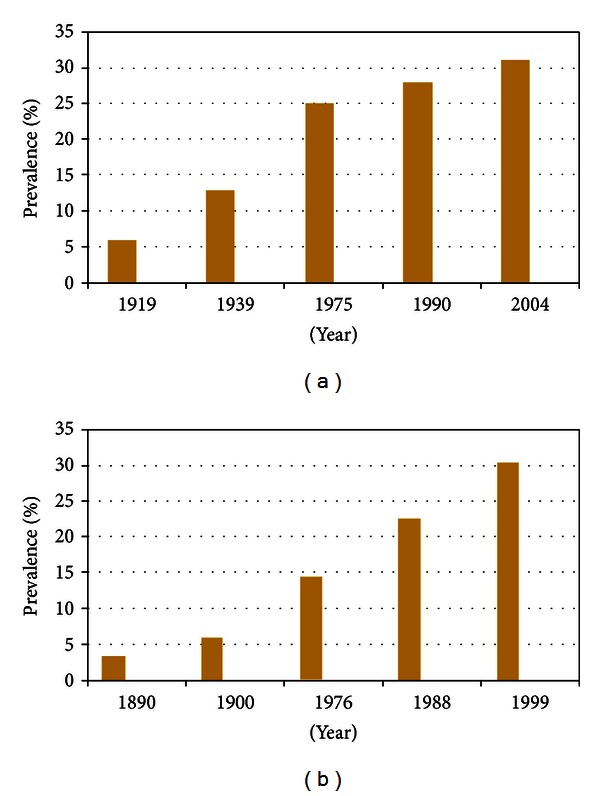
Changing prevalence of hypertension over 100 years in the USA (a). Increasing prevalence of obesity in the USA over 100 years. Obesity defined as body mass index (BMI Kg/m^2^) >30 (b).

Hypertension, diabetes, and obesity were originally documented in England and France where sugar first became available to the public. The rise in sugar intake in the United Kingdom and the United States also correlated with the rise in obesity rates observed in these countries [7]. In the early 1900s, blood pressure in over 140,000 healthy adults who applied for life insurance in the New York region suggested that a blood pressure of 140 (systolic)/90 (diastolic) mm Hg was abnormal because it reflected only 5-6% of the population in the United States [7]. Subsequent studies over the past century showed a significant and dramatic rise in the prevalence of hypertension in the United States [72–74] (Figure 6(a)). This was paralleled by an increase in the rates of obesity and diabetes. Body mass index (BMI; in kg/m^2^) >30 was observed in only 3.4% of 50- to 59-year-old male veterans in 1890, compared with 30.4% in 1999–2002 [7] (Figure 6(b)). This is paralleled by the epidemic of sugar consumption that has worsened over the past 300 years.

During the last decade, emerging data have altered our perspective on the link between fructose, uric acid, and hypertension. Epidemiologic data, in the form of large, longitudinal studies strengthened the link between elevated uric acid and hypertension [50, 75–79]. In animals, mild hyperuricemia induced by the uricase inhibitor oxonic acid, mimicking levels in humans, increased blood pressure by crystal-independent mechanism resulting in stimulation of the renin-angiotensin system and inhibition of nitric oxide synthase [80]. Two-thirds of adolescents with newly diagnosed essential hypertension and elevated uric acid normalized their blood pressure when treated with the xanthine oxidase inhibitor allopurinol [81]. This study could not exclude the possibility that some or all of the observed effect could have been mediated by a reduction in superoxide production, a byproduct of xanthine oxidase function. A more recent study done by the same group confirmed these results by using two differently acting urate lowering drugs, allopurinol, and probenecid [82]. This study clearly implicated uric acid as the biochemical mediator of increased blood pressure. Animal data suggested that uric acid induced hypertension has two phases. The first is salt insensitive which is likely to be managed by urate lowering drugs, while the second phase is salt sensitive. Due to a paucity of outcome data, recommendations on how to treat uric acid associate hypertension cannot be made at this time although the mechanism appears clear, especially in the early stages before the development of salt sensitivity. Future clinical trials are required to include different levels of hypertension and different age groups before recommending urate lowering agents especially as they have an inferior efficacy profile when compared with antihypertensive medications presently in clinical use.

Consumption of high-fructose chow by mice produced nocturnal hypertension and autonomic imbalance which may be related to activation of the sympathetic and RAS systems [83]. Subsequent data suggested that changes in autonomic modulation may be an initiating mechanism underlying the cluster of symptoms associated with cardiometabolic disease [84]. The addition of clonidine to drinking water inhibited fructose-induced hypertension in rats [85].

## 6. Effect of Fructose on Dyslipidemia and Insulin Resistance

The earliest recorded metabolic perturbation resulting from fructose consumption is postprandial hypertriglyceridemia, which increased visceral adipose deposition. Visceral adiposity contributes to hepatic triglyceride accumulation, protein kinase C activation, and hepatic insulin resistance by increasing the portal delivery of free fatty acids to the liver. With insulin resistance, VLDL production is upregulated and this, along with systemic free fatty acids, increase lipid delivery to muscle. It is also possible that fructose initiates hepatic insulin resistance independently of visceral adiposity and free fatty acid delivery [20]. Splanchnic perfusion studies have shown that hepatic production of triglycerides is much greater with fructose compared with equimolar concentrations of glucose [86]. Unlike glucose, fructose does not stimulate insulin secretion, due to its hepatic metabolism and the low level of expression of the fructose transporter GLUT5 in pancreatic *β*-cells [87]. Consumption of fructose-sweetened beverages with meals produced a rapid and prolonged elevation of plasma triglycerides compared with glucose-sweetened beverages. Because insulin, leptin, and possibly ghrelin function as key signals to the central nervous system in the long-term regulation of energy balance, decreases of circulating insulin and leptin and increased ghrelin concentrations could lead to increased caloric intake and ultimately contribute to weight gain and obesity during chronic consumption of diets high in fructose [88]. Apolipoprotein B levels were found to be higher following fructose consumption compared with isocaloric amount of glucose [89].

Fructose increases the incidence of hypertension, NAFL, and diabetes [90]. In fact, countries electing to use HFCS in their food supply have a 20% higher prevalence of diabetes compared to countries that did not use HFCS independent of obesity [91]. Uric acid stimulates fructokinase and the development of NAFL [14] via an increase in fructose metabolism thereby increasing the development of type 2 diabetes in children. This may be related to an increase in SREBP-1c and reduced acyl-CoA oxidase during pregnancy [92]. Body size at birth is related to food intake and the content of fructose [93] or an elevation of estrogen during pregnancy in women with a family history of type 2 diabetes [94, 95]. Genetic morphism in the glucokinase regulatory protein, which binds to glucokinase and inhibits its activity in the presence of fructose-6-phosphate (F6P) is associated with ethnicity and may be responsible for different response rates to obesity and diabetes in different populations [96]. An increase of fructose-1,6-biophosphatase [97], or in angiotensin 1–7 [98] decreases fructose-mediated diabetes by an increase in pancreatic islet metabolism [99].

## 7. Prospective and Therapeutic Implications

Based on the present knowledge of fructose and its detrimental metabolic effects when in excess and the unique nature of KHK, it is clear that fructose is, at least, partially responsible for the pandemic of diabetes and the metabolic syndrome that is presently occurring. A number of therapeutic approaches appear viable as a result of the data outlined previously.

(1) Assessing KHK activity in human blood samples opens a new approach to the diagnosis as well as the treatment of type 2 diabetes. We hypothesize that individuals either completely or partially deficient in KHK activity are immune at variable levels from developing type 2 diabetes mellitus. In order to test this hypothesis, the following require clarification: (a) KHK-C is primarily expressed in the liver and kidney. Thus, the *in vivo* handling of fructose to assess enzyme expression and efficiency would be difficult to monitor. We propose that PMN expression will parallel hepatic expression, a series of pilot clinical studies in individuals selected across populations at varying risk for type 2 diabetes mellitus is required. (b) Once this association has been demonstrated, measurement of KHK-C activity in PMN's in young adults with type 2 diabetes and age/sex matched controls is required. If results support the hypothesis, new approach to the prediction as well as a therapeutic approach to treat type 2 diabetes mellitus is at hand.

(2) Interference with fructose transport at the GLUT transporter level diminishes the availability of fructose. Due to their hydrophilic nature, sugars must first traverse lipid bilayer membranes via carrier-mediated transport mechanisms. Several transporters have been identified, and these include the facilitative glucose transporter (GLUT) which primarily regulates the clearance of blood glucose along a concentration gradient and the sodium-coupled glucose cotransporter (SGLT) family members that are distinct at both the primary and secondary structural levels from the GLUT proteins. Expression of SGLT proteins is restricted to the gut and kidney, where their role is energy-dependent reabsorption of glucose from lumen. The GLUT transporter family comprises 13 members that exhibit tissue distribution, substrate specificity, and transport kinetics that reflect their physiologic role. These have not been fully defined for all 13 isoforms [100]. Fructose gains access to the circulation and hepatocytes via GLUT 5, GLUT 7, and GLUT 11 [8]. Interference with fructose transport at the level of these transporters represents a therapeutic approach to prevent fructose-induced adiposity and insulin resistance.

(3) Interference at the level of KHK is an attractive approach to modify the metabolism of fructose and, possibly, alleviating adiposity and vascular dysfunction. This can be implemented in two ways. (a) An inhibitor is currently being developed (Richard Johnson, patent number WO/2012/019188, public knowledge). It remains unclear how effective this approach will be, and if this intervention will be dependent on an individual's KHK activity profile as suggested previously. (b) The use of certain furanose sugars, which can also be substrates for KHK and compete with fructose for its metabolism to hexose-1-phosphate [21]. D-Tagatose, a furanose sugar metabolized by KHK, when compared with fructose, caused markedly higher levels of serum uric acid and lower of Pi [101]. This highlights the importance of the poorly regulated KHK in this pathway and necessitates further study.

(4) Therapeutic agents that decrease the effect of high fructose diets on diabetes and insulin resistance include statins [102], metformin [103], and renin inhibitor attenuated diabetes and insulin resistance [104]. The latter is due to a decrease in lipid peroxidation. In support of these observations, candesartan cilexetil [105] and losartan improve renal hemodynamic and insulin resistance [106] and lower blood pressure in diabetic rodents fed a high fructose diet [107] presumably by the activation of AMP-activated protein kinase [108]. Infusion of Ang II decreased adiponectin and potentiated fructose-mediated insulin resistance in fructose-fed rats [109]. The presence of renin-angiotensin aldosterone in adipose tissue has been described [110–112] in which Ang II increased NADPH oxidase activity and WAT mediated inflammation and blocked RAAS thereby preventing the onset of diabetes. Blocking ROS via the inhibition of Ang II converting enzyme reduced obesity in rats [113] and dysregulation of inflammatory cytokines released by adipocytes [114, 115]. Plasma Ang II is associated with markers of insulin resistance and obesity [116]. Renin angiotensin expression regulates mouse and human adipocyte differentiation [117, 118].

(5) Uric acid can be a byproduct of uncontrolled fructose metabolism due to the rapid consumption of ATP as noted previously. Uric acid has been linked to endothelial and adipocytes dysfunction. In contrast, uric acid also functions as an antioxidant [119]. Treating hyperuricemia in the absence of gout is not recommended at this time due to the lack of sufficient data.

(6) The ideal and logical, but most difficult, approach would be to modify fructose content in food. The reason that KHK is a poorly regulated enzyme may be due to the high levels of physical activity and limited fructose intake that was prevalent in humans before the introduction of refined cane sugar and high fructose corn syrup (HFCS). Negative feedback was unnecessary due to the limited supply of fructose. This does not exist today where calorie sweeteners, including HFCS, are part of the normal diet. Moreover, and of grave concern, is the increased consumption of sugar-sweetened beverages, and fresh and processed juices which provide an easy vehicle for excessive sugar intake, over very a short period of time and have been directly linked to obesity [35, 120] and type 2 diabetes [45, 121]. At the end of the day, we might conclude that “only drinking “milk and water” can prevent diabetes.” The present choice is either to continue with the currently high rates of fructose consumption which lead to adipocyte expansion, obesity, and hypertension or to minimize fructose intake and alleviate health care cost and the cost of managing hypertension and its complications, thereby improving the lots of health care professional, patients, and their families and the well-being of millions of individuals.

## 8. Conclusion

Fructose metabolism is very unique in a sense that it is not regulated. The consequences of uncontrolled fructose metabolism can be harmful at the cellular level resulting in intracellular ATP depletion, increased uric acid production, endothelial dysfunction, oxidative stress, and increased lipogenesis. High fructose consumption induces insulin resistance and other manifestations of metabolic syndrome in a series of animal models. These effects are not seen in animals fed either glucose or starch. Human epidemiological data are generally of poor quality due to the lack of consistency in study design, methodology, and length. It remains unclear if targeting fructose by interfering with its transport or metabolism can be of any clinical benefit.
